# Tpeak-Tend ECG Marker in Obesity and Cardiovascular Diseases: A Comprehensive Review

**DOI:** 10.1155/2024/4904508

**Published:** 2024-06-26

**Authors:** Irena Dykiert, Kamila Florek, Krzysztof Kraik, Paweł Gać, Rafał Poręba, Małgorzata Poręba

**Affiliations:** ^1^ Division of Pathophysiology Department of Physiology and Pathophysiology Wroclaw Medical University, Wrocław, Poland; ^2^ Students' Scientific Association of Cardiovascular Diseases Prevention Department of Internal and Occupational Diseases Hypertension and Clinical Oncology Wroclaw Medical University, Wrocław, Poland; ^3^ Division of Environmental Health and Occupational Medicine Department of Population Health Wroclaw Medical University, Wrocław, Poland; ^4^ Department of Internal and Occupational Diseases Hypertension and Clinical Oncology Wroclaw Medical University, Wrocław, Poland; ^5^ Department of Paralympic Sport Wroclaw University of Health and Sport Sciences, Wrocław, Poland

## Abstract

Globally, cardiovascular diseases are still the leading cause of death. Numerous methods are used to diagnose cardiovascular pathologies; there is still a place for straightforward and noninvasive techniques, such as electrocardiogram (ECG). Depolarization and repolarization parameters, including QT interval and its derivatives, are well studied. However, the Tpeak-Tend interval is a novel and promising ECG marker with growing evidence for its potential role in predicting malignant arrhythmias. In this review, we discuss the association between the Tpeak-Tend interval and several cardiovascular diseases, including long QT syndrome, cardiomyopathies, heart failure, myocardial infarction, and obesity, which constitutes one of the risk factors for cardiovascular diseases.

## 1. Introduction

Cardiovascular diseases (CVDs) are among the most important and growing healthcare problems. They are the leading cause of death worldwide. In 2019, according to WHO data [1], nearly 17.9 million people died due to CVDs, and they accounted for almost 1/3 of all deaths worldwide. Due to this, research has been conducted to evaluate tools for finding pathology early or helping prevent sudden cardiac death (SCD) and other serious events. For this reason, there is still a search for novel and noninvasive or less invasive tools to detect and eventually monitor cardiovascular pathologies effectively.

Electrocardiography is a technique that is used to visualize and assess the heart's electrical activity. Information obtained with this test is helpful in the diagnostics of heart diseases, including arrhythmias, ventricular hypertrophy, ischemia, and myocardial infarction (MI). An electrocardiogram (ECG) may also be useful in detecting asymptomatic disorders, e.g., long QT syndrome (LQTS). Moreover, ECG has an application in risk stratification, monitoring of cardiac conditions, and response to treatment. ECG stands out from other tests mainly due to its safety, noninvasive character, easy and quick testing, and low cost. ECG can be widely used in almost every healthcare facility.

Numerous markers have been extracted from the ECG record, which reflects the various stages of the heart's function. Among them, there is a need to search for markers reflecting the myocardial depolarization and repolarization associated with malignant ventricular arrhythmias or predicting sudden cardiac death [2]. Some of these parameters, e.g., QT interval and calculated consistent with Bazett's formula-corrected QT interval (QTc), are commonly used to determine the risk of malignant arrhythmias. Other novel ECG markers, like Tpeak to Tend interval (Tp-e) and its derivatives, are still not used routinely.

The novel repolarization markers include Tpeak-Tend dispersion, JTpeak/JT, Tp-e/JTpeak, and Tpeak/JT ratios [3]. The JTpeak interval reflects early repolarization, while the Tp-e reflects late repolarization. Despite numerous studies, there is still uncertainty regarding the clinical application of the new parameters. Furthermore, no normal reference values of Tp-e and parameters and ratios are associated with that interval [4].

Describing the physiological role of the new marker Tp-e is an index of the transmural dispersion of ventricular repolarization, and it reflects the different durations of the action potential in the epicardium, endocardium, and M cells from the heart. As explained by Castro-Torres, the cellular mechanisms are translated to the T wave on the surface 12-lead electrocardiogram and allow the determination of an increase in the transmural dispersion of the ventricular repolarization through a measure from the peak or nadir to the end of the T wave. The Tp-e/QTc ratio includes values of ventricular repolarization's transmural and spatial dispersion [5].

**Figure 1:**
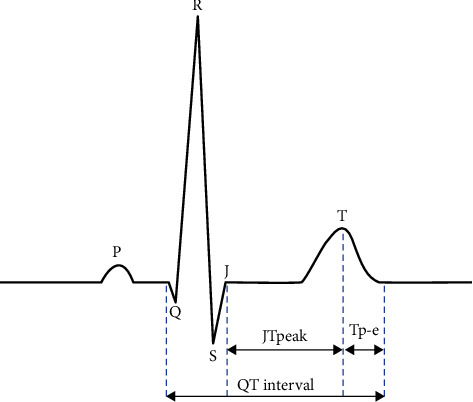
ECG repolarization intervals: QT, JTpeak, and Tp-e.

As new knowledge about QT interval, Tp-e, and other repolarization markers is still increasing, comprehensive reviews are needed to summarize the current knowledge about their role and the circumstances influencing their variations. This study aims to discuss the current state of knowledge and the most recent studies regarding the meaning and application of the Tp-e interval in selected disease entities and its predictive value. It also aims to identify gaps and inaccuracies in the current knowledge regarding this parameter (Figure 1).

## 2. Methods

### 2.1. Search Strategy

The review was conducted by searching PubMed, Embase, and Cochrane Library databases using keywords: Tpeak-Tend, Tp-e, TpTe, obesity, long QT syndrome, cardiomyopathy, heart failure, and myocardial infarction.

### 2.2. Inclusion and Exclusion Criteria

The inclusion criteria were (I) Tp-e-related studies; (II) disease associations with Tp-e among studies; (III) English language; (IV) publication in peer-reviewed journals; (V) adult population; and (VI) human population. We excluded animal studies, conference proceedings, case reports, and abstracts without complete text publication.

### 2.3. Literature Selection

**Figure 2:**
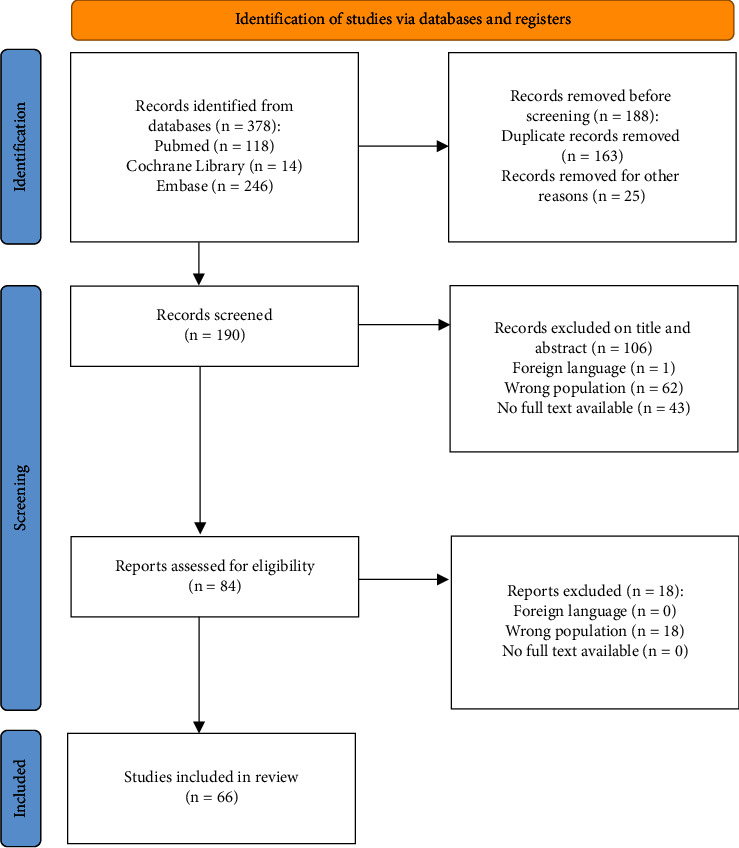
PRISMA flowchart for study selection.

We assessed the most relevant randomized controlled trials, case-control, cohort studies, and meta-analyses published between 2017 and 2023. Two reviewers independently evaluated three hundred seventy-eight articles according to the title, abstract, text, and scientific validity. As reported in the PRISMA flowchart, after removing duplicates (*n* = 163), 190 studies were initially screened, and 84 were found appropriate for a full assessment. In the end, 66 articles fulfilled the inclusion criteria (Figure 2).

## 3. Results and Discussion

### 3.1. ECG Parameters in the Prediction of Malignant Arrhythmias

The parameter considered, especially in the past, useful in predicting ventricular arrhythmias, including ventricular fibrillation (VF), is QT dispersion (QTd). QTd is defined as a difference between the longest and the shortest QT in the specific lead. Although QT interval has been confirmed to correlate with ventricular arrhythmias (VA), QT dispersion ranges vary widely between 10 and 71 ms, which may cause difficulties in its proper assessment [6]. QTd appears unreliable, even when appropriately measured, and is being questioned in extensive prospective studies [7]. Moreover, the correct measurement of QT dispersion is difficult and time-consuming. Other parameters have not yet been sufficiently researched for general use. Furthermore, it is believed that QT dispersion has been shown to reflect not the extent of heterogeneity of ventricular repolarization itself but the spatial position of the vectorcardiographic T loop [8].

Other markers can be used to assess heart function. Heart rate turbulence (HRT) is used to detect the impairment of the autonomic system and baroreflex, and it also has a prognostic role in assessing the risk of all-cause or sudden death [9]. HRT includes two parameters: turbulence onset and turbulence slope. Turbulence onset is a percentage of the length change between the mean of 2 RR intervals before and 2 RR intervals after ventricular premature beat. The turbulence slope is the steepest slope of the regression line of 5 consecutive RR intervals in the range of 15 RR intervals after ventricular premature beat.

Heart rate variability (HRV) is another marker used in cardiovascular risk stratification. It is measured as a change in the length of consecutive RR intervals. HRV is used to detect autonomic system dysfunction [10]. This method uses two HRV types: time-domain analysis and frequency domain (spectral). Both have been widely evaluated in the last decades. However, they are not routinely used in clinical settings as different studies are rather diverse. Secondly, markers of HRV are very dependent on sympathetic and parasympathetic balance. However, this analysis is applied in numerous 24-hour Holter systems. It was observed that HRV in frequency domain analysis was changed toward the sympathetic predominance in people with higher values of premature ventricular contractions in Holter monitoring, simultaneously in patients with higher blood pressure [11]. In the study by Cosgun and Oren authors compared repolarization markers: T-wave peak-end interval (Tp-e), QT, corrected QT (QTc), Tp-e/QT, Tp-e/corrected QT, and heart rate variability values in healthy men and women and to investigate their daily variations [12]. There were statistically significant differences in Tp-e and cTp-e intervals at various hours of the same day in both groups (women and men). In addition, there were statistically substantial moderate negative correlations between Tp-e intervals and SDNN at various hours of the same day. Some experts propose in this view that T peak-end markers should probably be adjusted to heart rate or use ratios such as Tp-e/QT and Tp-e/QTc, which is a better tool, independent from any changes in RR cycles and heart rate. This question should be studied in the coming years. Additionally, it should be commented on that the Tp-e/QTc ratio remains relatively constant between a heart rate of 60 and 100 beats/min. However, some researchers have recently published good outcomes after the correction of this parameter by the heart rate [13, 14].

Akdi et al. compared two groups of patients with higher and lower numbers of premature ventricular contractions in 24-hour Holter monitoring, and no significant differences were found in HRV time-domain indices. However, the study revealed that the Tp-e interval and Tp-e/QT are associated with the frequency of PVCs. HRV reflects the other type of physiological balance, that is, in the case of T peak indices and their derivatives [15]. In the different studies by Cosgun and Oren, including 500 healthy males categorized by five age subgroups, it occurred that there were significant differences between these groups in repolarization parameters in terms of Tp-e interval but not Tp-e/QT and Tp-e/QTc ratios. Considering the HRV parameters, there were statistically significant differences between the five male healthy groups in terms of HRV temporal parameters and no significant differences in HRV frequency parameters. The authors concluded that as the age increases, basal Tp-e interval increases and HRV temporal parameters decrease significantly in the male subjects aged between 30 and 79 years, but HRV frequency parameters do not change. The relations between the markers from the repolarization group and heart rate variability seem complex and need further evaluation [16].

T-wave alternans (TWA) is another marker with potential clinical use. TWA is the difference between consecutive T-wave amplitude and morphology. It may be helpful in the assessment of the risk of lethal ventricular arrhythmias and death due to cardiovascular events. Eventually, more studies are needed to confirm its usefulness, and this method requires more sophisticated equipment [17]. In 2008, in the AHA/ACCF/HRS Scientific Statement, TWA was listed as one of the potentially valuable markers of SCD risk due to a moderate amount of data. However, the measurement of TWA requires proper heart rate and regular RR interval duration. It was often inaccurate for many reasons, including failure to achieve the appropriate heart rate during gradual exercise and arrhythmias [18]. In most recent ventricular arrhythmias guidelines of AHA and ESC, it has no significant role [19, 20]. In ESC Guidelines, TWA is only used in modified LQTS diagnostic scores. Positive TWA adds 1 point to the score, requiring >3 points to diagnose LQTS [20].

Until now, QT interval has been well studied. There are commonly known factors influencing its duration, such as heart rate, age, hormone concentration, time of the day of the examination, potential imbalances in water volume and electrolyte concentrations, the influence of medications, and autonomic system nervous tension. In addition, essential QT interval changes may be observed in patients with heart failure and other heart diseases [21]. In contrast to QT or JTpeak intervals, studies claim that Tp-e duration is independent of HR [22]. However, it has been previously proposed that it be corrected with Bazett's formula, so it remains equivocal. Some studies reveal the Tp-e dependence of HR and suggest the Tp-e/QT ratio to be more appropriate in repolarization characteristics due to minimizing HR-influenced alterations [14]. In addition, Tp-e time may vary in different ECG leads [22].

The Tpeak-Tend interval (Tp-e) can be measured in many ways. Tp-e can be measured in a single lead or from the earliest Tpeak among all leads to the latest Tend throughout all leads. The second method is often used in research studies [23–25]. Moreover, the measurement of Tp-e in multiple leads is preferred to measurement in single lead because it allows the calculation of Tp-e dispersion, which is a difference between the highest and the lowest value of Tp-e in all leads [26]. Such an approach yields better results, especially when local changes are expected in myocardial ischemia. In one of the studies, the authors compared single-lead and multilead measurements of Tp-e and presented findings in favor of multilead measurements. Automated measurement is the most accurate Tp-e measurement method; among the manual methods, the tangent method is the most useful. The lead optimal for this measurement is the lead V2 [27].

Tp-e interval is the ECG parameter representing the dispersion of repolarization across the ventricles [28]. Notably, the predictive role of Tp-e prolongation and occurrence of SCD have been found, for example, in Oregon Sudden Unexpected Death Study [29]. The authors proved that prolonging the Tp-e interval was independently associated with SCD, with particular utility when the QTc was normal or not measurable because of prolonged QRS duration. In another study, the Tp-e interval is associated with SCD in adults with congenital heart diseases [30]. The potential mechanisms underlying the alterations visible in ECG as a Tp-e prolongation leading to the SCD may be driven by abnormal ion channel function, pathophysiological dispersions of repolarization providing a substrate for reentrant arrhythmias or autonomic dysfunction [31]. As suggested by Vehmeijer et al., various studies have demonstrated that there may be the transmural dispersion of repolarization due to prolonged repolarization of the subendocardial M cells or overall dispersion of repolarization [23, 30, 32].

Several authors discuss the importance of several parameters in repolarization characteristics, including Tp-e prolongation, compared to classical CVD risk factors, such as diabetes mellitus, hypertension, and smoking [33–35]. Moreover, obesity and structural and ischemic heart diseases also impact myocardial repolarization heterogeneity [36–38].

### 3.2. Tp-e Interval in Overweight and Obese People

Obesity is a disease characterized by excessive adipose tissue deposition in the body [39]. Obese individuals are more susceptible to developing cardiac diseases and dying of cardiovascular disorders, including SCD. Many changes indicate heart disease found more often in ECG records of obese people than in ECG records of people with normal weight, including prolongation of QT and QTc interval, prolongation of QT or QTc dispersion, tachycardia and higher heart rate, atrial and ventricular enlargement, conduction defects, left axis deviation, features of ischemia, old infarction, and repolarization abnormalities [40–42]. Moreover, obesity was associated with changes in the QRS complex in premenopausal women [43]. In children, obesity is associated with prolonged QT interval, longer QRS complex, and leftward shifts in frontal P-wave, QRS, and T-wave axes [44].

Several studies examining the connection between obesity and repolarization markers such as Tp-e, Tp-e/QT, and Tp-e/QTc support this association. Inanir et al.'s study [45] found that previously mentioned repolarization parameters, Tp-e/JT and Tp-e/JTc, were significantly increased in patients with class 3 obesity (body mass index (BMI) ≥40) in comparison with patients with normal body weight. Furthermore, according to Bağcı et al.'s research [46], Tp-e, Tp-e/QT, and Tp-e/QTc increase gradually with the growth of BMI. The association was found by examining these parameters in four groups of patients: normal weight (BMI: 18.0–24.9), overweight (BMI: 25–29.9), obese (BMI: 30.0–39.9), and class 3 obese (BMI ≥ 40). The same study found that Tp-e length was significantly positively correlated with age and systolic and diastolic blood pressure. This study suggests that repolarization impairment occurs before reaching class 3 of obesity.

On the contrary, Al-Mosawi et al. [47] associated the prolongation in repolarization markers (Tp-e, Tp-e/QT) with pericardial fat volume measured by multidetector computer tomography. They found no significant association between them and the growth of BMI. They found a significant association only in the group of patients with coronary atherosclerosis. In contrast to other studies, the Tp-e value decreased sequentially in groups of normal weight, overweight, and obese patients. The Tp-e interval was the shortest in the group of obese people, whereas, in most other studies, the obese have the most prolonged Tp-e interval. The result of this study may seem controversial because of this negative correlation. Some recent studies evaluated how weight loss due to bariatric surgery and sleeve gastrectomy surgery affected the change in markers of repolarization.

In trying to explain the conflicting results, it should be taken into account that both cardiac and extracardiac factors matter in the case of surface ECG. There may be a potential effect of extracardiac factors such as subcutaneous fat, heart position, fluid overload, and body habitus on the temporal parameters, not only on voltage parameters, which cannot be excluded entirely [48]. Generally, Tp-e is thought to reflect dispersion of repolarization, and this is an intracardiac factor. However, it should be considered that both obesity and weight loss may involve a change in extracardiac factors. This consideration might account for conflicting results.

It is also worth noting that values of Tp-e and its derivatives change after surgical procedures, leading to weight loss. Gul et al.'s study [49] included class 3 obesity patients and found a significant reduction in Tp-e, Tp-e/QT, Tp-e/QTc, Tp-e/JT, and Tp-e/JTc after bariatric surgery (measured 1 and 6 months after operation). The change in these parameters was also significantly correlated with weight loss. Ibisoglu et al. [50] also found a significant reduction in Tp-e, Tp-e/QT, Tp-e/QTc, Tp-e/JT, and Tp-e/JTc 6 months after bariatric surgery and stated that this change may reduce the risk of developing ventricular arrhythmia. Moreover, Inanir et al. [51] found that Tp-e interval, Tp-e/QT, Tp-e/QTc, Tp-e/JT, and Tp-e/JTc ratios decreased significantly after sleeve gastrectomy surgery in class 3 obesity patients, which suggests that weight loss by this surgery also reduces the risk of arrhythmias and SCD.

In conclusion, most studies support the statement that Tp-e duration and other repolarization parameters positively correlate with BMI. This indicates a higher risk of developing arrhythmias, including VAs, which may result in SCD, especially in obese patients. Opposing these findings, a study also shows the opposite trend: normal-weighted patients have the longest Tp-e, and obese patients have the shortest Tp-e. Moreover, studies agree that these parameters should be improved after weight loss due to bariatric surgery. However, only some studies examine the association between body weight and novel repolarization parameters, even though some are contradictory.

### 3.3. Tp-e Interval in Long QT Syndrome

LQTS is an electrical disorder that is characterized by the prolongation of QT interval [52] (QTc >440 ms in men and QTc >460 ms in women) [53]. LQTS increases the risk of polymorphic ventricular tachycardia (torsade de pointes), which might result in SCD. LQTS can be congenital or acquired. It may have an asymptomatic course in some people, and cardiac arrest may be the first sign of this disorder.

The length of the QT interval provides data about the time between depolarization and repolarization of cardiomyocytes. In addition to these data, the Tp-e interval includes information about the dispersion of repolarization. The Tp-e interval may be helpful in diagnostics and risk stratification in this condition.

Firstly, the Tp-e interval, along with other markers, may be used to determine whether the case of LQTS is congenital or acquired, which is essential because depending on the cause of the disease, the prognosis and treatment vary. Sugrue et al. [54] investigated T-wave morphology to differentiate whether LQTS is congenital or acquired. They found that patients with acquired LQTS had longer Tp-e in V5 lead than those with congenital LQTS. Patients with acquired LQTS also had a shallower right slope and a smaller T-wave centre of gravity. They suggested that T-wave morphology may also be useful in assessing IKr ion channel (a potassium channel that takes part in cardiac repolarization) activity in drug testing, especially in association with arrhythmogenesis. The Tp-e interval is a marker that appears to be effective in evaluating the risk of arrhythmogenesis, so the Tp-e interval could be one of the markers in this process.

Furthermore, Tardo et al.'s systematic review [55], apart from Sugrue et al.'s research, included Johannesen et al.'s [56] study, which found J-Tp and Tp-e intervals useful in the differentiation of IKr ion channel block and multichannel block in acquired LQTS. The block of IKr was associated with longer J-Tp and Tp-e intervals. The multichannel block (block of both IKr and calcium or sodium channel) was associated with shorter J-Tp and longer Tp-e and QT intervals.

Apart from differentiating congenital and acquired LQTS, Tp-e can potentially be used as a marker for assessing cardiac event risk in LQTS patients. Tse et al.'s meta-analysis [57] found that Tp-e is significantly longer in people with LQTS suffering from cardiac events than those without cardiac events and suggested that Tp-e may be useful in risk stratification. It was also found that the Tp-e/QTc ratio is also higher in high-risk patients, making it a useful risk marker. Furthermore, according to Markiewicz-Łoskot et al.'s study [58], Tp-e, combined with QTc, may have potential use in detecting affected relatives of people with congenital LQTS. They found that Tp-e is significantly longer in relatives affected by LQTS than in those unaffected and associated this marker with the possibility of cardiac events. They also found significant differences in Tp-e in LQTS type 1, LQTS type 2, and unaffected people when divided into groups based on sex. The Tp-e interval was also longer in LQTS type 2 than in LQTS type 1 without reaching statistical significance.

Moreover, according to Krych et al., longer Tp-e was associated with a higher risk of arrhythmia and cardiac events in people with LQTS type 7 (Andersen-Tawil syndrome). According to the same study, the higher value of Tp-e, QT, and U-wave presence in V2–V4 leads may also be related to the presence of KCNJ2 mutation [59]. Additionally, the meta-analysis by Tse et al. showed that the values of Tp-e and Tp-e/QT ratio among people with acquired QT interval prolongation were higher in patients with torsade de pointes incidents than in those without them, and due to that, these markers can be used in the stratification of risk in acquired LQTS [60]. Tp-e was longer in people with atrioventricular block-related LQTS and cardiac events than those without cardiac events, whereas Tp-e/QT was higher in people with drug-related LQTS and cardiac events than those without cardiac events.

In conclusion, the Tp-e interval may be a useful marker in differentiating congenital and acquired LQTS and assessing risk in LQTS independently from its cause.

### 3.4. Tp-e in Cardiomyopathies

According to ESC Guidelines, cardiomyopathy is a morphological and functional abnormality of the ventricular myocardium not caused by coronary artery flow limitation or abnormal loading conditions [61].

Furthermore, it is possible to distinguish five different subtypes of cardiomyopathy, each with genetic or nongenetic etiology. Those subtypes are hypertrophic cardiomyopathy (HCM), dilated cardiomyopathy (DCM), restrictive cardiomyopathy (RCM), arrhythmogenic right ventricular cardiomyopathy (ARVC), and the fifth, which consists of those unclassified [61].

According to HCM, the dominant cause of cardiomyopathy is sarcomeric protein gene mutation ranging about 40–60%, then 25–30% have unknown reasons, and 5–10% are other genetic or nongenetic causes, such as inborn errors of metabolism (e.g., glycogen storage diseases), neuromuscular diseases (e.g., Friedreich's ataxia), mitochondrial diseases (MELAS, MERFF), malformation syndromes (Noonan, LEOPARD, Costello, and CFC), amyloidosis, newborn of the diabetic mother, and drug-induced HCM [62].

Referring to SCD among HCM patients, there is a suggestion that Tp-e does not have a significant prognostic value in that group. However, QTc may be an appropriate tool for risk stratification in patients with HCM [63]. Another study revealed the utility of T-wave amplitude and traditional risk factors as an SCD marker in this group of patients. In contrast, this research did not show the statistical significance of Tp-e [64]. Importantly, in a large cohort of patients with HCM, it has been proven that a significant difference between genotype-positive and genotype-negative HCM patients in spatial mean and spatial peak QRS-T angles exists, which could be a better tool in identifying patients with HCM than traditional Seattle criteria. Moreover, Tp-e was significantly higher among genotype-positive patients than those without genetic backgrounds [65]. In addition, spatial QRS-T angle was shown to be significantly associated with VF and SCD in Brugada syndrome (BS) patients, who usually have structurally normal heart muscle; however, recently, the association between ARVC and BS molecular insight of pathogenesis was taken under scientific discussion [38, 66, 67]. The same study revealed that Tp-e does not have a predictive value in the BS group of patients according to malignant arrhythmias and SCD.

Dinshaw et al. showed that Tp-e prolongation in HCM patients is associated with VA, such as ventricular tachycardia (VT) and ventricular fibrillation (VF). It assessed its predictive role in SCD risk stratification. Besides, it is underlined that Tp-e < 78 ms among HCM patients with implanted ICDs is associated with a low risk of VA [67]. There were no significant differences among patients with transthyretin cardiac amyloidosis (TTR-CA) compared to the control group referring to Tp-e and Tp-e/QTc [68].

On the other hand, in the HCM population without a sarcoidosis background, Tp-e and Tp-e/QTc were significantly higher. They trended toward increased QT dispersion compared to the group without cardiac disease. The result present in the TTR-CA group is consistent with observed low-range SCD in that group of patients due to homogenous amyloid distribution [69]. Furthermore, the long-term prognostic value of ventricular repolarization dispersion in cardiac sarcoidosis patients was investigated. Endpoint was defined as the occurrence of the atrioventricular block (AVB), VT/VF, heart failure (HF) hospitalization, and all-cause death. That study assessed Tp-e/QT as an independent positive predictor of previously mentioned adverse events. VT/VF and SCD were observed more often in patients with greater Tp-e/QT ratios of ≥0.242 ms [70]. It was also confirmed that in patients without apparent heart involvement at early-stage sarcoidosis, QTcd, Tp-e, and Tp-e/QT ratios were significantly higher than in the control group [71].

Cardiomyopathy in Fabry disease is the most frequently present as left ventricular hypertrophic cardiomyopathy—in that group, ECG abnormalities included shorter P-wave and T-wave peak time, what was observed as a more symmetric T wave with lower T-wave time ratio described by Tonset-Tpeak/Tp-e compared to the control group [71]. There are contrasting results regarding the predictive value of Tp-e among HCM patients, especially its role in SCD prediction. The issue needs more studies and meta-analyses to assess its role in that population. In addition, a study confirmed that higher Tp-e among patients with nonischemic dilated cardiomyopathy was associated with malignant ventricular arrhythmias [72]. Lopez et al. investigated how ECG parameters predict SCD change after cardiac resynchronization therapy (CRT) with His bundle pacing. There was an improvement in QT interval, QT dispersion, and Tp-e dispersion, and Tp-e was shortened [73].

The study of Ponnusam et al. also noticed a significant improvement in repolarization parameters after His bundle pacing using QTc, Tp-e, and Tp-e/QTc ratio parameters. Those results prove a reasonable option for CRT among patients with left bundle branch block (LBBB)-induced cardiomyopathy with an improvement visible as normalization of electrical and mechanical pathologies [74]. However, in a group of HF patients with implantable cardioverter-defibrillator (ICD), in which 85.5% of patients (*n* = 272) were diagnosed with dilated cardiomyopathy, postimplantation Tp-e was revealed as an independent predictive factor of VT, VF, and all-cause mortality [75]. According to ARVC, one study corresponds to this review criterion, which revealed the association in longitudinal follow-up poorer prognosis and fragmented QRS, longer Tp-e in lead V2, and definite ARVC [76].

ESC Guidelines currently describe unclassified cardiomyopathies in Takotsubo cardiomyopathy and left ventricular noncompaction. Those pathologies found their association with ECG parameters. Patients with Takotsubo syndrome who suffered from major adverse cardiovascular events (MACE) (defined as acute heart failure, cardiogenic shock, sustained ventricular tachycardia, ventricular fibrillation, and death) had more often ST-segment elevation and their Tp-e/QT ratio was significantly higher. Tp-e/QT range >0.27, accompanied by low ejection fraction (EF), was defined as the subpopulation at higher risk of MACE [77].

When considering left ventricular noncompaction cardiomyopathies, patients compared to groups with normal cardiac ultrasound, Tp-e, and Tp-e/QT ratio were revealed as potential risk markers of that pathology presentation. These may be helpful markers before invasive procedures such as cardiac biopsy [78].

In conclusion, the role of Tp-e and its derivatives in assessing patients with cardiomyopathy may vary between its subtypes according to malignant ventricular arrhythmias. However, it can be an attractive parameter for monitoring a patient's prognosis after electrotherapy devices or LVAD implantation procedures. Furthermore, Tp-e can be a potential ECG marker, allowing preliminary diagnosis according to HCM genetic background and noncompaction cardiomyopathy suspicion before performing the cardiac biopsy.

### 3.5. Tp-e Interval in Heart Failure

Heart failure (HF) is when the cardiac output is reduced, and the heart cannot pump enough blood to meet the body's needs. The prevalence of HF grows rapidly in many countries because of the aging society. UpToDate shows about 64 million people worldwide suffered from this condition in 2022 [79]. A report from the American Heart Association estimated that the lifetime risk of heart failure development is 20–45% of people over 45, depending on the racial and ethnic group. Many circulatory diseases, including hypertension or coronary heart disease, may cause this condition. Heart failure increases the risk of death and reduces the quality of life [80].

In recent years, Piccirillo et al. conducted several studies [81–85] on patients suffering from heart failure and assessed the role of repolarization markers, including Tp-e, in chronic heart failure. There were attempts to find the use of repolarization markers in predicting hospital length of stay and the mortality of patients with acute decompensated heart failure [81]. It has been found that mean Tp-e was helpful as the predictor of mortality in the next 30 days, while Tp-e variance normalized and Tp-e standard deviation (Tp-eSD) was useful as the predictor of length of hospital stay and thus a predictor of severity. It was also suggested that these markers may be used to monitor both morphological and structural alterations of the heart.

It was also found that in patients with heart failure with a reduced ejection fraction (HFrEF), mean Tp-e and its standard deviation were higher than in those with heart failure with preserved ejection fraction (HFpEF) [82]. However, Son et al. found no difference in Tp-e and Tp-e/QT ratio based on ejection fraction in three groups of heart failure patients (preserved, midrange, and reduced ejection fraction) [86].

Mean Tp-e was also significantly correlated with mortality [81]. Patients who responded to the heart failure therapy had reduced Tp-eSD compared to nonresponders. Moreover, people with higher Tp-eSD died more often. In another study, mean Tp-e was related to chronic heart failure mortality, and Tp-eSD was a risk factor for aggravation and complications of this disease [83]. Another Piccirillo et al. study found an association between Tp-e and mortality in acutely decompensated chronic heart failure [84]. In a study including patients with decompensated heart failure and atrial fibrillation (AF), mean Tp-e was again associated with mortality, while higher Tp-eSD was associated with permanent AF [85]. The important conclusion from the last study is that Tp-e is not affected by atrial fibrillation.

Several recent studies have investigated repolarization markers in heart failure therapies. Usalp et al. investigated the length of Tp-e and T wave in cardiac resynchronization therapy. They found that a reduction in the duration of these markers was a significant predictor of a favorable response to the treatment [87]. Moreover, Banavalikar et al. found that Tp-e predicts ventricular tachyarrhythmias in heart failure patients after cardiac resynchronization therapy [88]. Furthermore, Li et al. investigated the association between left bundle branch area pacing (LBBAP) and echocardiographic response in heart failure patients. They found that Tp-e is useful as the predictor of response to the therapy, especially in patients without left bundle branch block [89]. Patients with Tp-e shorter than 81.2 ms after therapy were significantly more likely to be responders than those with longer Tp-e. They also found that Tp-e interval duration and Tp-e/QTc ratio were reduced considerably after therapy in patients with QRS >130 ms. Also, Xue et al. investigated Tp-e in patients with heart failure and ICD and its use in predicting VA and mortality [75]. It was found that a longer duration of Tp-e was positively associated with VT, VF, and mortality.

In conclusion, the Tp-e interval and its derivatives may be practical in predicting mortality and severity during heart failure. Moreover, it may be used to monitor the effectiveness of therapy. It should be kept in mind that most of the studies conducted in recent years have been carried out by one research team, and there is a need to replicate these studies in other populations.

### 3.6. Tpeak-Tend in Myocardial Infarction

Tpeak-Tend interval and Tpeak-Tend/QT ratio were essential predictors among patients with myocardial infarction (MI), mainly in those with ST-segment elevation myocardial infarction (STEMI). However, groups with myocardial infarction with nonobstructive coronary artery disease (MINOCA) were investigated according to repolarization parameters, including Tp-e.

Prolonged Tp-e was confirmed to be an independent risk factor of VA in STEMI patients after percutaneous coronary intervention (PCI) [90, 91]. In addition, the Tp-e interval measured before the procedure was found to be an independent predictor of reperfusion VF [92]. One study did not confirm the Tp-e association with VF, as it was statistically insignificant among other investigated parameters [93]. Patients described with MINOCA had a significantly higher risk of VA, and Tp-e Tp-e/QT was longer in that group [94]. According to myocardial reperfusion, Tp-e >72.5 ms and Tp-e/QT ratio >0.18 independently predicted procedure impairment, in-hospital MACEs, and poorer 6-month survival rate [93]. Moreover, the 3-year survival rate among patients with prolonged Tp-e/QT in infarct-related leads corresponded with patients' higher mortality [95].

Namazi et al. retrospectively confirmed the statistical significance of QT dispersion, Tp-e value, and in-hospital mortality in STEMI patients with those parameters measured before PCI [96]. In addition, Tp-e was revealed to be an independent predictive factor of incomplete ST-segment resolution in STEMI patients treated with PCI [97]. However, there was a trial analyzing only patients with acute anterior MI. Its results contradicted those previously mentioned, revealing no statistical significance of Tp-e or Tp-e/QT in patients undergoing PCI [96]. Moreover, Wang et al. assessed Tp-e as an independent predictive factor of 1-year MACE defined as cardiac death and malignant arrhythmia event [90].

An analysis according to preinfarction angina (PIA) was conducted and revealed that STEMI patients with PIA had a lower chance of suffering from VA than those without PIA–who had longer Tp-e, Tp-e/QT ratio, which independently predicted in-hospital VA [98]. Interestingly, Tp-e was found to predict Intensive Care Unit (ICU) stay among patients with acute coronary syndrome (ACS) with COVID-19 [99].

Tp-e prolongation was also associated with the marker of coronary artery disease (CAD) severity [100]. One of those markers is the SYNTAX score; its higher scores were associated with prolonged Tp-e and Tp-e/QT in patients with CAD [101–103]. These results may be an interesting potential noninvasive predictive marker of CAD severity.

In conclusion, those findings may help invasive cardiologists stratify the procedural and periprocedural risk, including VA, MACE, mortality, and long-term prognosis.

### 3.7. Tpeak-Tend in Valvular Heart Disease

Changes in Tp-e length and derived parameters have been reported in valvular heart diseases. Most studies focus on the relationship between Tp-e and its derivatives and aortic stenosis (AS). Patients with aortic stenosis have significantly higher values of Tp-e interval, Tp-e/QT, and Tp-e/QTc ratios than healthy people [104]. The more severe the AS, the more significant the parameter increase. Moreover, a positive correlation was found between the Tp-e/QTc ratio and the mean aortic gradient.

Furthermore, the Tp-e/QTc ratio was a significant and independent predictor of severe AS. Another study observed increases in Tp-e interval, Tp-e/QT, and Tp-e/QTc ratios [105]. In this study, again Tp-e/QT and Tp-e/QTc ratios were significantly associated with AS, and mean aortic gradient was positively correlated with Tp-e, Tp-e/Qt, Tp-e/QTc, and Tp-ed. Also, a negative correlation between aortic valve areas and Tp-e, Tp-e/Qt, Tp-e/QTc, and Tp-ed was found.

In several studies, after transcatheter aortic valve implantation (TAVI), there was a significant reduction in values of Tp-e, Tp-e/Qt, Tp-e/QTc, and Tp-ed, indicating that TAVI might reduce the risk of ventricular arrhythmias and mortality [105, 106]. Moreover, before TAVI, there was a positive correlation among Tp-e, Tp-e/QT, Tp-e/QTc, and left ventricular mass index (LVMI) [106]. Tp-e was also independently associated with LVMI.

Another study found Tp-e and its derivatives useful in predicting complete atrioventricular blocks after TAVI [107]. Tp-e, Tp-e/QT, Tp-e/QTc, Tp-e/JT, and Tp-e/JTc were significantly higher in patients requiring permanent pacemaker after TAVI and, additionally, Tp-e/QTc and Tp-e/JTc were significantly associated with the presence of complete atrioventricular block. Moreover, Tp-e/JTc was a potential independent predictor of complete atrioventricular block after TAVI.

Tp-e, Tp-e/QT, Tp-e/QTc, and Tp-ed also had higher values in the group of patients who died after successful treatment of AS with surgical aortic valve replacement (SAVR) in comparison with patients who survived within a mean follow-up period of 66.3 ± 42.4 months [108]. Tp-e, Tp-e/QT, Tp-e/QTc, and Tp-ed were independent mortality predictors after SAVR. Higher values of Tp-e/QT and Tp-e/QTc were associated with a lower chance of long-term survival. In another study, lower values of preprocedural Tp-e were associated with better survivability after TAVI [109]. The follow-up period in this study was one year.

Contrary to most previously mentioned studies, Chino et al.'s study showed no significant changes in Tp-e, Tp-e/QT, and Tp-e/QTc after neither TAVI nor surgical aortic valve replacement [110].

Similarly to patients with aortic stenosis, the patients with severe mitral stenosis have significantly higher values of Tp-e interval, Tp-e/QT, and Tp-e/QTc ratios than the healthy population [111]. Moreover, after percutaneous mitral balloon valvuloplasty, the value of these parameters significantly decreased, revealing the beneficial effect of this procedure on the mentioned repolarization parameters. Furthermore, the Tp-e interval, Tp-e/QT, and Tp-e/QTc ratios also had higher values in children with mitral valve prolapse when compared to healthy children [112]. Moreover, the value of Tp-e/QTc correlated positively with the degree of mitral regurgitation. It is suspected that these changes might be associated with the increased risk of ventricular arrhythmias and SCD in patients with mitral valve prolapse.

In conclusion, most studies suggest that Tp-e and its derivatives have increased values in valvular heart disease, and the proper treatment of this disease might reduce the values of these parameters. The high values of these parameters are also associated with a worse prognosis after treatment of valvular disease.

### 3.8. Tpeak-Tend in Brugada Syndrome

Patients with Brugada syndrome (BS) are more susceptible to ventricular arrhythmia and sudden cardiac death if the Tp-e interval is prolonged [113]. In BS, the ajmaline challenge is performed to provoke BS, and there was research investigating whether prolonged Tp-e or the corrected interval can predict the positive result of the ajmaline challenge. However, its role declined [114]. Other studies revealed a significant correlation between Tp-e prolongation and the occurrence of life-threatening arrhythmic events among patients with BS [115, 116].

Importantly, Tp-e was found to be the most promising ECG marker together with QTc interval in predicting malignant arrhythmias among BS patients. However, there is a need to assess the cut-off for the Tp-e value, which significantly increases the risk of life-threatening arrhythmias [117]. Interestingly, when the BS typical ECG pattern was observed in precordial leads V1–V3, it was simultaneously revealed that the Tp-e interval in V1 lead was significantly higher among patients with malignant arrhythmias. In contrast, in the other leads, no significant differences were noted [113]. However, in the bigger cohort, it was observed that among BS patients in the mean follow-up of 88 months, Tp-e was not significantly prolonged in those with syncope or malignant arrhythmias [118].

A consistent statement on the role of the Tp-e interval in risk stratification in BS should be evaluated in further research. The pathophysiological mechanism is still under debate with the considered potential mechanisms including the “depolarization hypothesis” and “early repolarization hypothesis” [119].

Similarly, as in BS, in patients with J wave syndrome who were aborted from sudden cardiac death, the Tpeak-Tend interval and Tp-Te/QT ratio are significantly increased [120].

### 3.9. Other Cardiovascular Diseases and Sleep Apnea

The role of Tp-e has been noted concerning various clinical conditions. Importantly, acute myocarditis (AM) is one of these conditions, and prolonged Tp-e, Tp-e/QT, and Tp-e/QTc ratios have been observed in patients with AM [121]. What makes an interesting insight into the AM patient's characteristics in terms of ECG changes as usually observed abnormalities are sinus tachycardia or nonspecific ST-T wave changes [122]. However, elucidating the patients' prognosis revealed in the Ucar et al. study parameters among AM patients would make a practical application of these results [121].

Tp-e characteristics in hypertension have been confirmed to be prolonged in nondipper hypertension and positively correlated with the cardio-ankle vascular index in this population [123, 124]. Tp-e and Tp-e/QTc parameters in patients with arterial hypertension increased in patients with subclinical myocardial dysfunction diagnosed by the echocardiography parameter, left ventricular global longitudinal strain (LV-GLS) [125].

Tp-e and its derivatives were essential parameters in patients with hypothyroidism, where they were prolonged in both overt and subclinical presentations [126, 127]. As the cardiovascular system is significantly affected in liver cirrhosis, it has been revealed that heart rate, Tp-e, Tp-e/QT, and Tp-e/QTc were considerably higher in the diseased group than in the control group [128]. Moreover, the same study showed the predictive value of heart rate, Tp-e, and Tp-e/QT for end-stage liver cirrhosis, although no correlation with Child stages was observed. Another study confirmed that Tp-e, QTc interval, Tp-e/QTc ratio, and fQRS are increased in liver cirrhosis, noting a parallel association of these parameter prolongations with disease severity [129]. However, this contradicted the findings of the previously mentioned study, as it showed a significant correlation with the Pugh-Child classification.

Obstructive sleep apnea (OSA) is a risk factor for ventricular arrhythmias. Assessing which patients are at a higher risk of this complication is important, as demonstrated in Yan et al.'s study [130]. According to their retrospective analysis, patients who presented nocturnal premature ventricular contractions had a significantly higher Tp-e/QT ratio than those with OSA. Another study conducted among patients with OSA revealed a significant correlation between moderate and severe OSA and increased Tp-e, Tp-e/QT, and Tp-e/QTc ratios [131]. However, findings describe Tp-e, Tp-e/QT, and Tp-e/QTc prolongation only during the apnea period, with a decrease in the postapnea hyperventilation period [132].

Another disease confirmed to be correlated with altered repolarization is chronic obstructive pulmonary disease (COPD), where significant prolongation of Tp-e, Tp-e/QT, and Tp-e/QTc ratio compared to the control group was observed [133]. According to blood test results, it was revealed that low ferritin levels among female patients without anemia or history of cardiac disease, as well as vitamin B12 deficiency in the healthy adult population, influence their arrhythmogenic susceptibility, which was observed by increased Tp-e, Tp-e/QT, and Tp-e/QTc parameters [134, 135]. Moreover, the significantly higher values of Tp-e, Tp-e/QT, and Tp-e/QTc were also observed in patients with benign paroxysmal positional vertigo who were admitted to the emergency department when compared to the healthy population, suggesting that these patients might be prone to cardiac arrhythmias [136].

## 4. Conclusions

In conclusion, Tp-e and its derivatives are very promising ECG markers. Tp-e may be a potential marker in several groups of patients; in each, it may provide different vital clinical prognoses. It can be associated with the genetic background of certain diseases, e.g., LQTS or HCM. Moreover, its prolongation may help stratify patients' prognosis among patients with HF cardiomyopathy after several invasive management procedures and those with MI treated by PCI.

**Figure 3:**
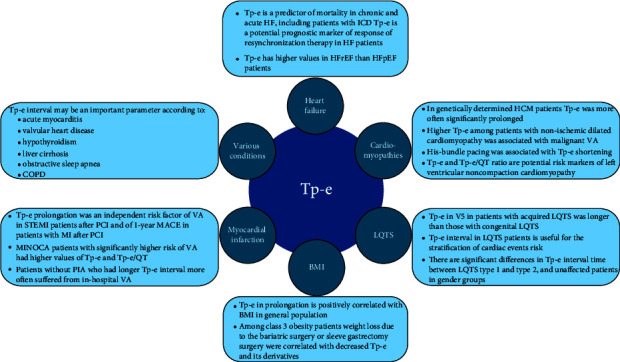
Tp-e interval: current state of knowledge summary.

In the case of obesity, studies considering conditions from normal weight through overweight and subsequent classes of obesity would be particularly valuable. The role of Tp-e in VA and SCD is visible, although situations should be evaluated in meta-analysis to reach a consensus on that issue. More randomized trials are needed to define the parameters influencing Tp-e duration and the value of its derivatives, and more studies are required to precisely evaluate the association between Tp-e and derived parameters of diseases examined in this study (Figure 3).

## Data Availability

No data were used to support this study.
